# Impaired cerebrovascular reactivity in sepsis-associated encephalopathy studied by acetazolamide test

**DOI:** 10.1186/cc8939

**Published:** 2010-03-31

**Authors:** Szilárd Szatmári, Tamás Végh, Ákos Csomós, Judit Hallay, István Takács, Csilla Molnár, Béla Fülesdi

**Affiliations:** 1 Department of Anesthesiology and Intensive Care, University of Debrecen, Health and Medical Science Center, H-4032. Debrecen, Nagyerdei krt. 98, Hungary; 2 1st Department of Surgery, Semmelweis University, H-1082 Budapest, Üllõi út 78, Hungary; 3 Department of Surgery, University of Debrecen, Health and Medical Science Center, H-4032 Debrecen, Nagyerdei krt. 98

## Abstract

**Introduction:**

The pathophysiology of sepsis-associated encephalopathy (SAE) is not entirely clear. One of the possible underlying mechanisms is the alteration of the cerebral microvascular function induced by the systemic inflammation. The aim of the present work was to test whether cerebral vasomotor-reactivity is impaired in patients with SAE.

**Methods:**

Patients fulfilling the criteria of clinical sepsis and showing disturbance of consciousness of any severity were included (n = 14). Non-septic persons whithout previous diseases affecting cerebral vasoreactivity served as controls (n = 20). Transcranial Doppler blood flow velocities were measured at rest and at 5, 10, 15 and 20 minutes after intravenous administration of 15 mg/kgBW acetazolamide. The time course of the acetazolamide effect on cerebral blood flow velocity (cerebrovascular reactivity, CVR) and the maximal vasodilatory effect of acetazolemide (cerebrovascular reserve capacity, CRC) were compared among the groups.

**Results:**

Absolute blood flow velocities after adminsitration of the vasodilator drug were higher among control subjects than in SAE. Assessment of the time-course of the vasomotor reaction showed that patients with SAE reacted slower to the vasodilatory stimulus than control persons. When assessing the maximal vasodilatory ability of the cerebral arterioles to acetazolamide during vasomotor testing, we found that patients with SAE reacted to a lesser extent to the drug than did control subjects (CRC controls:46.2 ± 15.9%, CRC SAE: 31,5 ± 15.8%, *P *< 0.01).

**Conclusions:**

We conclude that cerebrovascular reactivity is impaired in patients with SAE. The clinical significance of this pathophysiological finding has to be assessed in further studies.

## Introduction

Sepsis-associated encephalopathy is defined as a diffuse cerebral dysfunction induced by the systemic response to the infection without clinical or laboratory evidence of direct infectious involvement of the central nervous system [1]. Previous clinical observations have shown that the brain is often the first organ to be affected by sepsis, preceeding the clinical symptoms of other organ manifestations. According to the studies of Wilson and colleagues and Young and colleagues, electroencephalogram (EEG) may be abnormal in 87% of patients with bacteriemia. They diagnosed 70% with disturbance of consciousness of differing severity ranging from somnolence to coma [1-3]. Ebersoldt and colleagues, reviewing sepsis-associated delirium, reported on a prevalence ranging from 9 to 71% [4]. The exact pathomechanism involved is not yet fully understood. It is believed that microcirculatory alterations, disturbance of cerebral autoregulation, damage of the blood-brain barrier, branched chain/aromatic amino acid inbalance and the direct effect of the inflammatory process (e.g. free radicals, oxydative stress, cytokines, excitotoxicity apoptosis) on glial cells may play a decisive role. Sepsis-related encephalopathy is most likely to be a multifactorially determined syndrome [5].

When assessing cerebral microvascular contributing factors, in previous human investigations Matta and Stow [6] found cerebral autoregulation and carbon dioxide reactivity to be normal in patients with sepsis, whereas Terborg and colleagues reported on severely disturbed vasomotor reactivity (VMR) [7]. In the past two decades, different stimuli have been used to test cerebral autoregulation and metabolic regulation, such as altering arterial partial pressure of carbon dioxide (pCO_2_) either by inhalation of carbon dioxide or by changing respiratory rate (carbon dioxide reactivity), breath holding test (carbon dioxide reactivity), decreasing systemic blood pressure and therewith cerebral perfusion pressure (cerebral autoregulation) and intravenous injection of acetazolamide. Acetazolamide, the reversible inhibitor of the enzyme carbonic anhydrase, has been used to test cerebral VMR in various diseases and conditions [8]. Disturbed cerebrovascular reactivity (CVR) as a sign of cerebral microvascular alterations has been demonstrated in patients with diabetes mellitus [9,10], arterial hypertension [11], systemic lupus erythematosus [12], in subjects hemodynamically significant stenoses and occlusions of the carotid arteries [13]. With respect to the debated involvement of the above cerebral microvascular alterations, in the present study we intended to test whether acetazolamide-induced cerebral VMR is altered in patients with sepsis-associated encephalopathy. To the best of our knowledge this is the first study that uses the transcranial Doppler-acetazolamide test to assess cerebral VMR in sepsis-related encephalopathy.

## Materials and methods

The study was approved by the local Medical Ethics Committee of the Debrecen University Health and Medical Science Centre. Patients fulfilling the criteria of clinical sepsis according to the guidelines of the American College of Chest Physicians/Society of Critical Care Medicine (ACCP/SCCM) Consensus Conference Committee [14] were enrolled in the study. Those with hemodynamic instability, in need of hemodynamic support or with signs of hypoperfusion of the different organs were excluded. Patients were not under mechanical ventilation prior to or during the study. Patients were selected and screened during daily rounds on the postoperative surgical wards or from the multidisciplinary surgical ICU.

Sepsis-related encephalopathy was defined as a combination of the following: patients had to meet the criteria of clinical sepsis and had to show disturbance of consciousness or alertness of any severity. Any other metabolic causes of conscious disturbance were excluded (hypoxemia, hyper-or hypoglycemia, increased serum urea, creatinine or ammonia levels). A certified neurologist (BF) performed a detailed neurological assessment of all the patients in order to exclude direct infectious involvement of the central nervous system (such as meningitis or encephalitis). Sedative drugs were not administered before the neurological assessment. Consciousness/alertness disturbance was graded by two scales: the Richmond Agitation-Sedation Scale (RASS) and the Ramsay scores. The different categories of these scoring systems are described elsewhere in detail [15]. As septic patients suffered from altered consciousness, their nearest relatives were asked to give informed consent. When sepsis and encephalopathy were diagnosed, patients were transferred to the ICU and a continous monitoring of arterial blood pressure, echocardiography, pulse oxymetry was initiated. This made it possible to perform arterial blood gas analysis every five minutes after acetazolamide administration.

Transcranial Doppler measurements were performed in the supine position using a Rimed Digilite Transcranial Doppler sonograph (Rimed Ltd, Raanana, Israel). A 2 MHz probe was used for insonation, and sample volume, gain and power were kept constant during the investigation. Temporal window was used for insonation, probes were fixed by LMY-2 probe holder (Rimed Ltd, Raanana, Israel). The device enabled the assessment of the best available signal of the middle cerebral artery between the depths of 45 to 55 mm. Systolic, diastolic and mean blood flow velocities were registered, and pulsatility indices were calculated by the device. After a blood flow velocity measurement was performed at rest, 15 mg/kg acetazolamide (Diamox, Lederle Pharmaceuticals, Carolina, Puerto Rico, USA) was injected intravenously. As proposed in previous studies [8], blood flow velocities were continously registered until 20 minutes after injection of the vasodilatory stimulus. CVR was defined as the percentage increase of the middle cerebral artery mean blood flow velocity after administration of acetazolamide. CVR was calculated as follows:

where MCAV_ACZ _is the middle cerebral artery mean blood flow velocity measured at 5, 10, 15 and 20 minutes after acetazolamide, and MCAV_rest _is the middle cerebral artery mean blood flow velocity measured at rest. Cerebrovascular reserve capacity (CRC; the maximal percentage increase of the blood flow velocity after acetazolamide administration), was calculated as follows:

where MCAV_ACZmax _is the highest mean blood flow velocity in the middle cerebral artery within 20 minutes after administration of acetazolamide.

Transcranial Doppler measurements were performed in 20 age- and sex-matched persons, who were free of sepsis, diabetes mellitus, hypertension, significant stenoses of the cerebral arteries or any known diseases which, according to our present knowledge, could have influenced CVR testing. These subjects served as controls for the study. In these subjects arterial sampling for blood gas analysis was only performed at resting state, because inserting a radial artery catheter or serial arterial sampling during the whole study was considered unethical.

### Statistical analysis

Means and standard deviations were reported for all values. Before performing statistical comparisons of the parameters, a normality test was used. Parameters with normal distribution were compared with the appropriate unpaired t-tests. Repeated measure analysis of variance was used to detect differences in MCAV and CVR values after acetazolemide administration. When significant differences were detected, pairwise comparisons were performed between the groups using the Mann-Whitney U test. Differences were accepted as statistically significant if *P *value was less than 0.05.

## Results

Fourteen patients with sepsis-associated encephalopathy and 20 control persons were enrolled. Blood pressure values assessed by arterial blood pressure did not change during the acetazolamide testing. During the study, slight hyperventilation was observed, but any deterioration of the patients' status did not occur during or after acetazolamide. The results of the most important clinical and laboratory data of septic patients and controls are summarized in Table 1. From these data it can be seen that blood pressures and blood gas analysis parameters were comparable in the two groups at rest. In septic patients, pH slightly decreased, while pCO_2 _and partial pressure of oxygen slightly increased during the acetazolamide test. The distribution of the Ramsay scales were in the septic groups as follows: Ramsay 1 = 6 cases, Ramsay 3 = 4 cases, Ramsay 4 = 4 cases. There were five cases with RASS +1 and a further eight cases with RASS -1. Thus, in all cases either a sepsis-related delirious state or somnolence was present.

**Table 1:** Results of the most important clinical or laboratory parameters before in septic and in control patients

	Sepsis	Control	*P *value
**Systolic BP (mmHg)**	117.9 ± 10.3	113.5 ± 8.7	0.20
**Diastolic BP (mmHg)**	69.7 ± 5.9	75.0 ± 5.4	0.01
**Mean BP (mmHg)**	84.7 ± 7.6	87.8 ± 5.3	0.21
**Arterial pH**			
0 minutes	7.39 ± 0.04	7.40 ± 0.03	0.48
5 minutes	7.38 ± 0.04	NA	-
10 minutes	7.37 ± 0.03	NA	-
15 minutes	7.37 ± 0.04	NA	-
20 minutes	7.37 ± 0.04	NA	-
**Arterial pCO_2 _(mmHg)**			
0 minutes	36.8 ± 3.4	38.9 ± 1.96	0.11
5 minutes	38.2 ± 3.5	NA	-
10 minutes	41.0 ± 4.4	NA	-
15 minutes	40.8 ± 3.9	NA	-
20 minutes	41.3 ± 4.9	NA	-
**Arterial pO_2 _(mmHg)**			
0 minutes	87.0 ± 9.7	83.7 ± 3.46	0.07
5 minutes	91.5 ± 11.2	NA	-
10 minutes	91.5 ± 9.3	NA	-
15 minutes	91.2 ± 8.9	NA	-
20 minutes	90.0 ± 9.0	NA	-
**WBC count (G/l)**	15.1 ± 6.4	5.93 ± 1.84	< 0.001
**PCT**	8.89 ± 8.7	NA	-

Means and standard deviations are shown.

BP: blood pressure; NA: not available; PCO_2_: partial pressure of carbon dioxide; PCT: procalcitonin; PO_2_: partial pressure of oxygen; WBC: white blood cell count.

**Figure 1:**
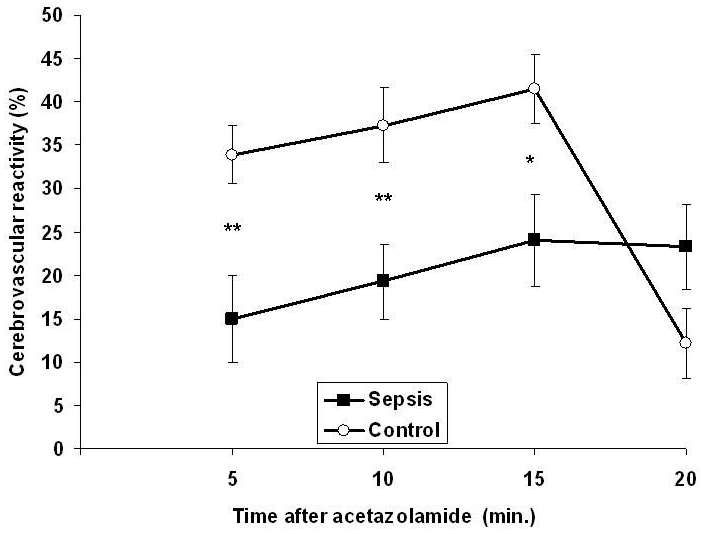
**Percentage increase of the middle cerebral artery mean blood flow velocity in patients with sepsis-associated encephalopathy and in controls at 5, 10, 15 and 20 minutes after injection of acetazolamide**. Means and standard errors are shown.

**Figure 2:**
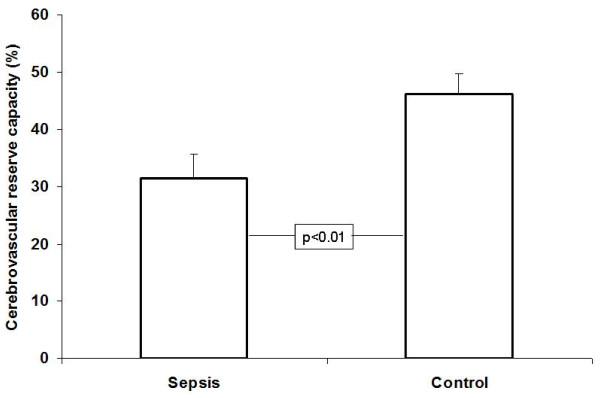
**Maximal percentage increase of the middle cerebral artery mean blood flow velocity in patients with sepsis-associated encephalopathy and in controls after injection of acetazolamide**. Means and standard errors are shown.

The results of the transcranial Doppler measurements are summarized in Table 2. Resting systolic blood flow velocities did not differ, but the mean and the diastolic blood flow velocities were lower in the group with sepsis-associated encephalopathy. It has to be noted that pulsatility indices were higher at the resting state in patients with sepsis-related encephalopathy and this difference remained unchanged after administration of acetazolamide. Absolute blood flow velocities after the vasodilator drug were higher among control subjects than in septic patients. In a further analysis we checked the time-course of the vasomotor reaction to acetazolamide. As shown in Figure 1, patients with sepsis-associated encephalopathy reacted slower to the vasodilatory stimulus than control persons. When assessing the maximal vasodilatory ability of the cerebral arterioles to acetazolamide during 20 minutes of vasomotor testing, we found that patients with sepsis-associated encephalopathy reacted to the drug to a lesser extent than control subjects. The results are depicted in Figure 2.

**Table 2:** Systolic, diastolic and mean blood flow velocities (cm/s) and pulsatility indices before and after administration of acetazolamide in control persons and in patients with sepsis-associated encephalopathy

Time after acetazolamide (minutes)	Sepsis (n = 14)	Control (n = 20)	*P *value
Systolic blood flow velocity			
**0**	85.4 ± 20.7	85.9 ± 13.7	0.94
**5**	99.6 ± 31.6	114.1 ± 20.5	0.15
**10**	96.5 ± 24.2	118.5 ± 19.5	< 0.05
**15**	101.9 ± 27.1	124.4 ± 17.5	< 0.05
**20**	102.0 ± 27.7	121.9 ± 17.4	< 0.05
**Diastolic blood flow velocity**			
**0**	32.5 ± 12.3	45.6 ± 8.8	< 0.01
**5**	35.9 ± 12.5	61.9 ± 12.6	< 0.001
**10**	40.1 ± 13.3	64.2 ± 13.9	< 0.001
**15**	43.2 ± 17.4	64.4 ± 11.7	< 0.001
**20**	40.0 ± 12.6	80.4 ± 14.3	< 0.001
**Mean blood flow velocity**			
**0**	47.9 ± 14.5	58.2 ± 12.0	< 0.05
**5**	55.4 ± 18.2	77.8 ± 17.1	< 0.01
**10**	56.4 ± 16.0	79.3 ± 16.6	< 0.001
**15**	59.4 ± 19.4	64.4 ± 11.7	< 0.01
**20**	58.7 ± 17.5	80.4 ± 14.3	< 0.001
**Pulsatility index**			
**0**	1.15 ± 0.35	0.85 ± 0.20	< 0.01
**5**	1.21 ± 0.26	0.80 ± 0.16	< 0.001
**10**	1.01 ± 0.32	0.70 ± 0.16	< 0.01
**15**	0.98 ± 0.34	0.76 ± 0.15	< 0.05
**20**	1.06 ± 0.24	0.74 ± 0.14	< 0.01

Means and standard deviations are shown.

## Discussion

In the present study we found that cerebral VMR is impaired in patients with sepsis-associated encephalopathy. It is also clear from our results that not only maximal vasodilative capacity (CRC) but also the time-course of the vasodilative effect (CVR) is affected after administration of acetazolamide in septic patients. Thus, the reaction of the cerebral arterioles to the vasodilatory stimulus is not only lower in magnitude, but also occurs slower in patients with sepsis-associated encephalopathy.

When analyzing absolute blood flow velocities in the middle cerebral artery, it is clear that they are lower in patients with sepsis-associated encephalopathy compared with non-septic control persons after acetazolemide stimulation. A decrease in the blood flow velocity measured within the middle cerebral artery may theoretically be explained in two ways: either the large and medium-size vessel (the middle cerebral artery) is dilated or there is a vasoconstriction at the level of resistance arterioles of its corresponding territory. Although this question cannot be answered based only on the absolute blood flow velocity values, taking the pulsatility indices into account, the higher pulsatility index among patients with sepsis-associated encephalopathy is more likely to indicate vasoconstriction of the cerebral arterioles. It has been shown previously that an increase in resistance distal to the site of insonation results in an increased blood flow pulsatility [16]. Thus, based on our results, decreased cerebral blood flow velocities along with higher pulsatility indices in patients with sepsis-associated encephalopathy can be ascribed to the vasoconstriction of the resistance arterioles. These results are in accordance with previous studies stating that cerebral blood flow is reduced and cerebrovascular resistance is increased in sepsis-associated encephalopathy [1,17]. It seems that general vasodilation does not affect the brain circulation in sepsis; instead a vasoconstriction of the resistance arterioles occurs. This is the explanation for the findings of Matta and Stow, who found that sepsis-induced vasoparalysis does not involve the cerebral vasculature [6].

There are numerous factors in sepsis that may contribute to the vasoconstriction of the brain resistance arterioles. First, in animal experiments it has been demonstrated that the blood-brain barrier, which normally maintains a homeostatic environment for brain cells, becomes leaky within the first hours of endotoxemia. Disruption of the blood-brain barrier allows high levels of endogenous catecholamines to directly influence cerebrovascular resistance [18]. Second, it is believed that cytokines and ILs produced during the course of the sepsis cascade may alter the activity of the endothelial nitric oxide synthase. The inhibition of endothelial nitric oxide synthase leads to the impairment of the microcirculation of the brain by causing vasoconstriction [1]. Finally, alterations of the coagulation system resulting in microthromboses and microinfarctions as seen in sepsis may also contribute to the microvascular dysfunction [19].

The goal of cerebral autoregulation and metabolic vasoreactivity testing is to see whether the brain circulation is able to adopt to sudden and critical changes of blood pressure (autoregulation) or metabolic demands (metabolic regulation). From the previous clinical investigations and animal experiments it is clear that cerebral arterioles of 40 to 200 μm in diameter are common actors of both autoregulatory and metabolic response of the brain circulation. Different stimuli have been used to test cerebral autoregulation and metabolic regulation, such as altering pCO_2 _(carbon dioxide reactivity), breath holding test (carbon dioxide reactivity), decreasing systemic blood pressure and therewith cerebral perfusion pressure (cerebral autoregulation) and intravenous injection of acetazolamide. Basically, there are two main factors to take into account during VMR tests: the maximal vasodilative capacity (CRC) and the time-course of the reaction (CVR) [8]. In the present study we used intravenous acetazolamide to assess the cerebral vasomotor response.

**Figure 3:**
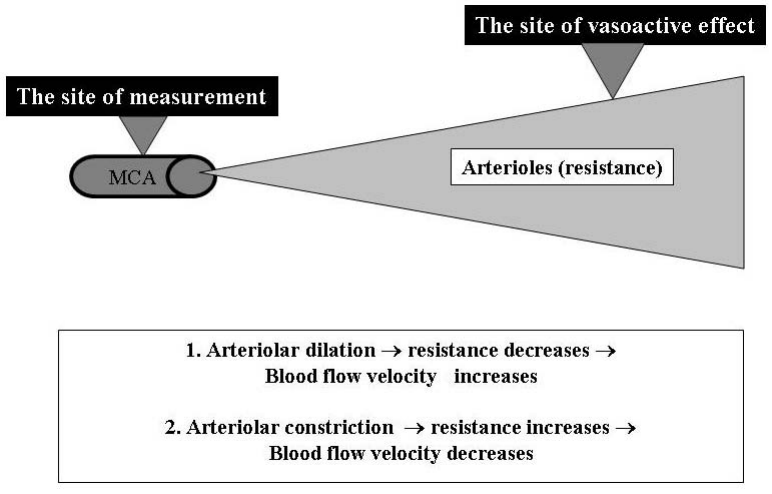
**Illustration of the rationale and the background of transcranial Doppler-assessed cerebral vasomotor reactivity testing**. MCA: middle cerebral artery.

For the sake of clarity we intend to explain the concept of transcranial Doppler acetazolamide tests. Acetazolamide is a reversible inhibitor of the carbonic anhydrase, which is located at the surface of the erythrocytes. The enzyme catalyses the following reaction: CO_2 _+ H_2_O → H_2_CO_3 _→ H^+ ^+ HCO_3_). It also induces a slight temporary hypercapnia lasting for approximately 20 minutes, which results in vasodilation of the cerebral arterioles, most probably through inducing nitric oxide synthesis [8]. As described above, cerebral arterioles are key actors in cerebral autoregulation and metabolic regulation. Dilation of these vessels results in a decrease of cerebrovascular resistance. As shown in Figure 3, transcranial Doppler measurements can be performed at the level of the middle cerebral artery and cerebral arterioles cannot be directly assessed. When an arteriolar vasodilation occurs, the cerebrovascular resistance of the corresponding arterial territory decreases, resulting in an increase of the cerebral blood flow velocity measured in the middle cerebral artery. Thus, cerebral arteriolar function cannot be directly measured. Only changes of the cerebrovascular resistance induced by acetazolamide can be indirectly assessed by measuring cerebral blood flow velocities in the middle-sized arteries of the corresponding territory. It has to be noted that there are some limitations of our study. Transcranial Doppler does not measure cerebral blood flow. It measures cerebral blood flow velocity, the changes of which are not equal, but only proportional to changes of cerebral blood flow. A further limitation is the lack of arterial pCO_2 _monitoring in the control group.

In our study, a less intensive CVR was detected in patients with sepsis-associated encephalopathy, that is cerebral arterioles reacted to the vasodilator stimulus slower and to a lesser extent. Besides a slower vasodilation after acetazolamide administration, the maximal dilation of the cerebral arterioles (CRC) was also lower in septic patients. These results are in accordance with those of Terborg and colleagues, who also demonstrated dysfunction in patients with severe sepsis and septic shock [7]. Similarly, animal studies have showed decreased carbon dioxide-induced VMR in streptococcal sepsis [20]. In recent animal models it has been shown that microcirculatory dysfunction in the brain precedes changes in evoked potentials [21]. Taking the absolute blood flow velocities and pulsatility indices in the present study into account, it is conceivable that vasoconstriction of the cerebral arterioles may be responsible for the impaired VMR. As shown in Table 2, pulsatility indices were higher throughout the entire course of the acetazolamide test among septic patients compared with control persons, suggesting vasoconstriction of the resistance vessels. Although there was a slight difference between diastolic pressures of septic and control persons, it has to be noted that mean arterial pressures in the two groups were similar and therefore the significance of this BP difference during transcranial doppler sonography (TCD) -acetazolamide testing most probably did not influence the results.

## Conclusions

The clinical signficance of the present study may be summarized as follows. First, the results of the transcranial Doppler acetazolamide test may help to better understand the pathophysiology of septic encephalopathies. Second, as we mentioned above, cerebral autoregulation and metabolic regulation occur at the same level of the cerebral circulation (resistance arterioles). In our series of septic patients without hemodynamic compromise or need of hemodynamic support, the ability of the brain resistance arterioles to dilate was decreased. If it is considered that sepsis-associated shock situations and sudden decreases of cerebral perfusion pressure evoke a strong autoregulatory response, an already reduced vasodilatory capacity should limit both the static and dynamic autoregulatory response of the cerebral arterioles. One of the most important functions of cerebral autoregulation is to ensure constant cerebral blood flow (and therewith oxygen delivery) during changes in systemic blood pressure. Further studies are needed to clarify the importance of hemodynamic monitoring and proper hemodynamic support in early phases of sepsis (and sepsis-related encephalopathy is an early warning sign), in order to prevent critical blood pressure changes in the cerebral vascular bed and thus the progression of brain damage.

## Key messages

• Cerebral arteriolar function is altered in sepsis-associated encephalopathy

• Cerebral arterioles of patients with SAE react lesser extent to vasodilatory stimuli

• Cerebral hemodynamic changes may be involved in the early pathogenetic phases of SAE

## Abbreviations

CRC: cerebrovascular reserve capacity; CVR: cerebrovascular reactivity; ECG: echocardiogram; EEG: electroencephalogram; MCAV: middle cerebral artery mean blood flow velocity; PCO_2_: partial pressure of carbon dioxide; RASS: Richmond Agitation-Sedation Scale; VMR: vasomotor reactivity.

## Authors' contributions

SS and TV performed the transcranial Doppler tests. ÁC and MC participated in the design of the study. JH and IT drafted the manuscript. BF performed neurological examinations. BF and MC participated in planning the design of the study, performing the statistical analysis, and completing the manuscript. All authors read and approved the final manuscript.

## Competing interests

The authors declare that they have no competing interests.
